# Resolving the gene expression maps of human first-trimester chorionic villi with spatial transcriptome

**DOI:** 10.3389/fcell.2022.1060298

**Published:** 2022-12-06

**Authors:** Zhongzhen Liu, Man Zhai, Qingqing Zhang, Tingyu Yang, Zunmin Wan, Jianlin Li, Xiaofeng Liu, Bo Xu, Libei Du, Rachel W. S. Chan, Li Zhang, William S. B. Yeung, Ka Wang Cheung, Philip C. N. Chiu, Wen-Jing Wang, Cheuk-Lun Lee, Ya Gao

**Affiliations:** ^1^ BGI-Shenzhen, Shenzhen, China; ^2^ Department of Obstetrics and Gynaecology, LKS Faculty of Medicine, The University of Hong Kong, Pokfulam, Hong Kong SAR, China; ^3^ Shenzhen Key Laboratory of Fertility Regulation, The University of Hong Kong-Shenzhen Hospital, Shenzhen, China; ^4^ Shenzhen Engineering Laboratory for Birth Defects Screening, Shenzhen, China; ^5^ College of Life Sciences, University of Chinese Academy of Sciences, Beijing, China; ^6^ Department of Obstetrics and Gynaecology, The University of Hong Kong-Shenzhen Hospital, Shenzhen, China

**Keywords:** spatial transcriptomics, Stereo-seq, placenta, chorionic villi, spatial regulatory activity, virus receptors, drug transporters

## Abstract

The placenta is important for fetal development in mammals, and spatial transcriptomic profiling of placenta helps to resolve its structure and function. In this study, we described the landscape of spatial transcriptome of human placental villi obtained from two pregnant women at the first trimester using the modified Stereo-seq method applied for paraformaldehyde (PFA) fixation samples. The PFA fixation of human placenta villi was better than fresh villi embedded in optimum cutting temperature (OCT) compound, since it greatly improved tissue morphology and the specificity of RNA signals. The main cell types in chorionic villi such as syncytiotrophoblasts (SCT), villous cytotrophoblasts (VCT), fibroblasts (FB), and extravillous trophoblasts (EVT) were identified with the spatial transcriptome data, whereas the minor cell types of Hofbauer cells (HB) and endothelial cells (Endo) were spatially located by deconvolution of scRNA-seq data. We demonstrated that the Stereo-seq data of human villi could be used for sophisticated analyses such as spatial cell-communication and regulatory activity. We found that the SCT and VCT exhibited the most ligand-receptor pairs that could increase differentiation of the SCT, and that the spatial localization of specific regulons in different cell types was associated with the pathways related to hormones transport and secretion, regulation of mitotic cell cycle, and nutrient transport pathway in SCT. In EVT, regulatory pathways such as the epithelial to mesenchyme transition, epithelial development and differentiation, and extracellular matrix organization were identified. Finally, viral receptors and drug transporters were identified in villi according to the pathway analysis, which could help to explain the vertical transmission of several infectious diseases and drug metabolism efficacy. Our study provides a valuable resource for further investigation of the placenta development, physiology and pathology in a spatial context.

## Introduction

The placenta is important in fetal development of mammals by enabling exchange of gas, nutrients and wastes between mother and fetus. It secretes hormones and growth factors to facilitate fetal growth and development *in utero*. As the functional unit of human placenta, the chorionic villi has a complex and heterogeneous structure composing of different types of cells, including fibroblasts (FB), vascular endothelial cells (Endo), Hofbauer cells (HB), villous cytotrophoblast (VCT), syncytiotrophoblast (SCT), extravillous trophoblasts (EVT), and endothelial cells of blood capillary networks (Strauss, 1964). Depending on attachment to decidua, chorionic villi can be categorized as floating villi and anchoring villi. The multinuclear SCT are localized to the outermost layer of the villi and serve as a barrier between the mother and the fetus for the exchange of nutrients and gas (Aplin, 2010). The SCT also produce pregnancy hormones, including placental lactogen and human chorionic gonadotrophin (hCG), to regulate growth of the fetus and the placenta (Costa, 2016). The VCT are located beneath the SCT and proliferate to form the villi branches. The VCT can differentiate into the SCT and the EVT, the latter of which are localized to the distal cell column of anchoring villi (Knofler et al., 2019). The EVT migrate from these villi and invade the decidua to remodel the spiral arteries (Pijnenborg et al., 1980). The inner core of the villi contains a large proportion of fibroblast-like stromal cells (Ilic et al., 2008). The HB are fetal macrophages that are involved in placental vasculogenesis and development of the villous tree (Tang et al., 2011). All the above-mentioned cells exert different roles and interact with each other to ensure a healthy pregnancy outcome. Any developmental defects of the placenta may lead to pregnancy complications such as preterm birth, miscarriage, intrauterine growth restriction and preeclampsia (PE) (Pollheimer and Knofler, 2012), which are associated with long-term adverse outcomes in maternal and perinatal health.

Due to the importance of the placenta in fetal-maternal communications, development and functions of the placenta is a hot research topic for a long time. Early placental studies were mainly based on histological analysis of specimens (Hertig et al., 1956; Hamilton and Boyd, 1960), *in vitro* tissue culture (Deglincerti et al., 2016; Shahbazi et al., 2016) and animal models (Enders, 2007; Cockburn and Rossant, 2010). Recently, emerging technology of single-cell RNA sequencing (scRNA-seq) has greatly promoted placenta researches, including placental development at different gestational weeks (Liu et al., 2018; Suryawanshi et al., 2018; Vento-Tormo et al., 2018) (Tsang et al., 2017) and placental functions in pregnancy complications such as PE (Zhang et al., 2021a), preterm birth (Pique-Regi et al., 2019), and gestational diabetes mellitus (Yang et al., 2021). However, scRNA-seq could not be used to study *in situ* physical interaction and microenvironment because the spatial information of cells is lost during cell dissociation prior to scRNA-seq.

The next generation sequencing (NGS)-based spatial transcriptomics method was published in 2016, which allowed each transcript to be mapped back to its original location using unique positional molecular barcodes (Stahl et al., 2016). Although this method has been rapidly adopted in many cell atlas projects, there is still no report on the spatial transcriptome of early human chorionic villi. The recently developed Stereo-seq, using the random barcode-labeled DNA nanosphere microarray pattern deposition, presents higher resolution and larger field-of-view than previously reported spatial transcriptomics methods. This method has recently been used to establish spatial and temporal transcriptome profiles of mouse embryos, Drosophila embryos, zebrafish embryos, axolotl brain and Arabidopsis leaves (Chen et al., 2022a; Cheng et al., 2022; Jiang et al., 2022; Liu et al., 2022; Wang et al., 2022; Xia et al., 2022). In this study, we successfully constructed the spatial transcriptome of human placental villi using a modified protocol of Stereo-seq, which provides a valuable resource for future placental researches.

## Materials and methods

### Sample preparation

Two healthy pregnant women for termination of pregnancy (TOP) surgery at the eighth week due to psychosocial reasons were recruited in this study. The TOP surgery was performed at the Department of Obstetrics and Gynaecology, The University of Hong Kong-Shenzhen Hospital. Before the surgery, the participants received genetic counselling, and signed a consent form for the study, which was approved by the Institutional Review Board of The University of Hong Kong-Shenzhen Hospital ([2021]208) and the Institutional Review Board of BGI (BGI-IRB 22118).

The placentas were collected by vacuum aspiration. The villi samples were sorted immediately after the surgery based on the villous morphology. Fetal membranes and tissues with blood clots were discarded. About 1 cm × 1 cm × 1 cm of the fresh villi samples were washed in a 1.5 ml tube containing 1 × pre-cooled phosphate buffered saline (PBS) (GIBCO, 70011044, from 10 × PBS diluted with diethylpyrocarbonate treated water) on ice to remove the residual blood. The samples were then mixed with pre-cooled Tissue-Tek optimum cutting temperature (OCT) compound (Sakura, 4583), snapped frozen in liquid nitrogen and stocked at −80°C. All these procedures were completed within 30 min after sample collection.

**FIGURE 1:**
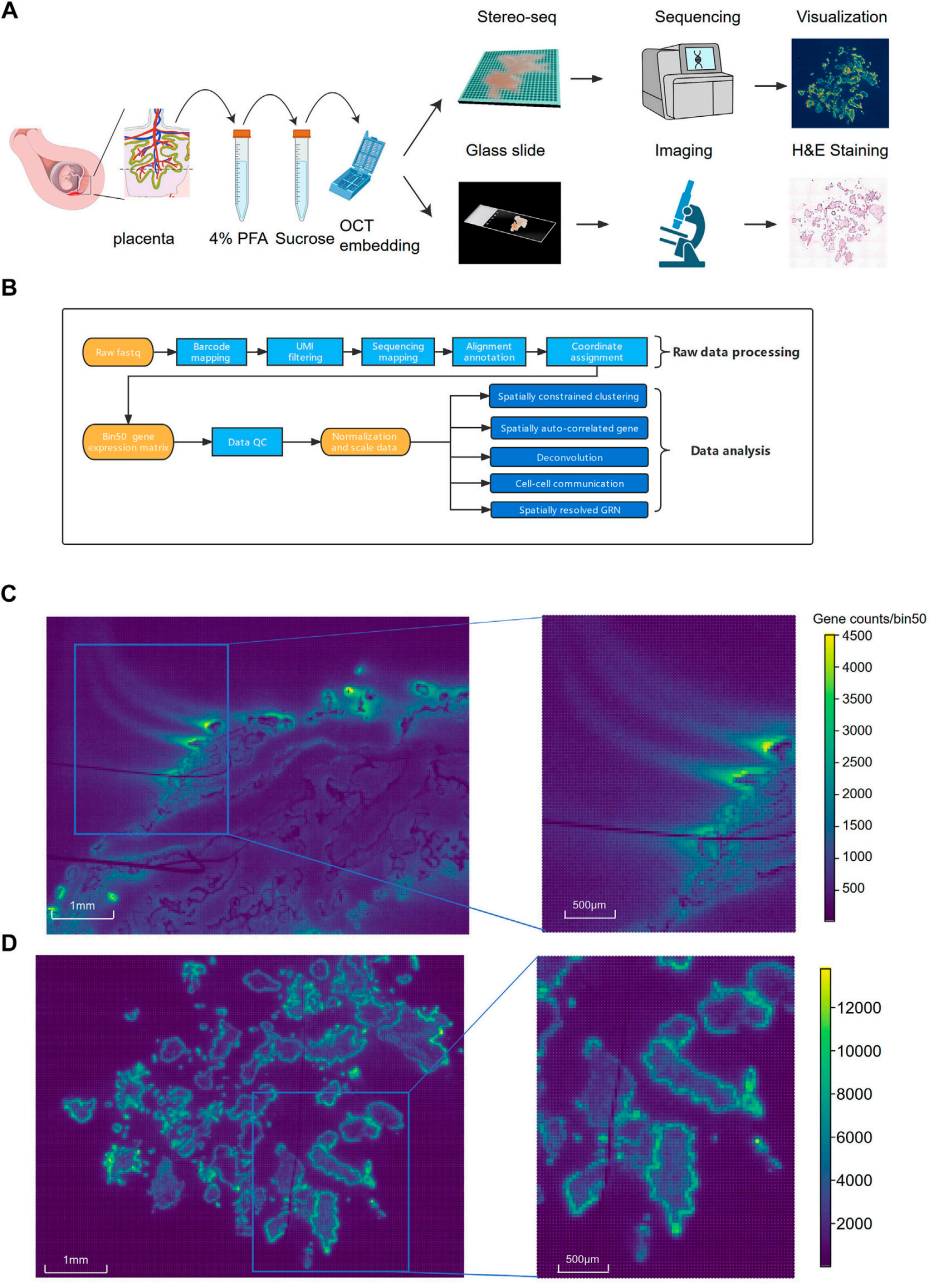
The schematic diagram of the experimental, data analysis workflow and comparison of Stereo-seq performance on fresh and PFA fixed villi samples. **(A)** The workflow of Stereo-seq using the PFA-fixed villi. **(B)** Pipeline of the data analysis. **(C)** The distribution of the signals on the chip from fresh villi sample. **(D)** The distribution of the signals on the chip from PFA-fixed villi.

For paraformaldehyde (PFA)-fixed samples, the villi were trimmed to less than 1 cm × 1 cm × 2 cm block, washed with PBS and immersed in 4% PFA (BOSTER, AR1069) at a 10:1 ratio of volume for 16 h at 4°C (Figure 1A). The fixed tissues were then dehydrated in 10% sucrose for 4–6 h, 20% sucrose overnight and 30% sucrose for 24 h at 4°C. The dehydrated samples were embedded in OCT as mentioned above. The fresh villi and the PFA-fixed samples were transferred in dry-ice. Cryosections were conducted sagitally at a thickness of 10 μm in a cryostat (DAKEWE Cryostat Microtome, 6250). Three successive cryosections of the largest intersecting surface were obtained from each sample. One of the cryosection was used for Hematoxylin and Eosin (H&E) staining and the other two sections were used for Stereo-seq as replicates.

### Stereo-seq libraries construction

For the OCT-embedded fresh samples, Stereo-seq libraries were prepared and sequenced using the STOmics Gene Expression kit S1 (BGI, 1000028493) following the standard protocol V1.1 as previously described (Chen et al., 2022a). Briefly, tissue sections were immediately adhered to a Stereo-seq chip at −20°C, and warmed by fingertip to melt the OCT. Then the chip was fixed in methanol for 30 min at −20°C. After fixation, single-strand DNA dye (Thermo fisher, Q10212) was added to image the nucleus under a Ti-7 Nikon Eclipse microscope. Then the permeabilization enzymes were added to release the transcripts under a condition with pH = 2. After reverse transcription, the residual tissues were digested, and the cDNA was released from the chips and purified using the AMPure XP beads (Vazyme, N411-03). The indexed Stereo-seq libraries were quantified by the Qubit ssDNA Assay Kit (Thermo Fisher Scientific, Q10212). The PFA-fixed samples were prepared with an extra de-crosslinking procedure by immersing the chips into the Tris(hydroxymethyl)aminomethane-ethylendiaminetetraacetic acid (Tris-EDTA) buffer (pH = 10) containing 5% RNase inhibitor at 70°C for 1 h before permeabilization. After de-crosslinking and permeabilization, the chips were re-fixed in pre-cooled methanol at −20°C for 15 min. The captured RNA were reversely transcribed to cDNA. After amplification, fragmentation and cyclization, the DNA nanoballs (DNB) were sequenced on a MGI DNBSEQ-T10 sequencer (MGI, Shenzhen, China) according to the manufacturer’s protocol (50 bp for read 1, 100 bp for read 2) (Chen et al., 2022a).

### Data processing and quality control

The fastq files containing sequenced reads generated by the MGI DNBSEQ-T10 sequencer were processed using the STOmics Analysis Workflow (SAW) v4.1.0 (https://github.com/BGIResearch/SAW) (Chen et al., 2022a) to generate spatial gene expression matrices (Figure 1B). The coordinate identity (CID) and the unique molecular identifiers (UMIs) sequences are in read 1 while the cDNA sequences are in read 2. Firstly, CID were mapped to the designed coordinates of the *in situ* captured chip, and low-quality sequences were filtered out based on the UMIs quality score. Then read 2 was aligned to the reference genome (hg38) using STAR (Dobin et al., 2013). Aligned reads with MAPQ < 10 were filtered out, and retained reads were further mapped to corresponding genes. After merging barcode reads count, a CID-containing expression profile matrix was generated for further quality control. The Stereo-seq raw data were analyzed at the resolution of bin50 (50 × 50 DNB, or 37.5 μm × 37.5 μm) in this study, with the transcripts from the same genes combined into an aggregated profile within each bin as described earlier (Chen et al., 2022a). First, the distributions of both log-transformed total counts and log-transformed n genes by counts were calculated, and the presence of bimodal distributions suggested strong background signals. After that, bin50s with the percent counts in top 100 genes reaching 100% were removed. The remaining bins were then clustered by using the Spatially Constrained Clustering (SCC). The clusters generated from background signals were filtered out. The Wilcoxon test was conducted to identify the differentially expressed genes (DEG). The dissociative counts from dead cells were filtered, since most of the top genes with high expression levels were mitochondrial and ribosomal genes. After filtering out the low-quality clusters, the final expression matrix was used for the downstream analysis.

### Spatially constrained clustering

Principal component analysis (PCA) of the expression matrix were conducted using the Scanpy package (Wolf et al., 2018). SCC was performed on the dimensional-reduced data with consideration of both gene expression similarities and neighborhood relations in spatial distribution. In brief, the spatial k-nearest neighbor (KNN) graph, produced by the Squidpy (Holgersen et al., 2021), was integrated with the KNN graph which was built from the transcriptomic data by the Scanpy. The graph including gene expression and spatial information was then passed to the Leiden algorithm for further clustering, and the markers of each output clusters were identified by the rank_genes_groups function of Scanpy with the Wilcoxon method. All clusters were annotated by combining the information from the marker genes and canonical markers of villi cell types.

### Spatially resolved gene regulatory network: SCENIC

The gene regulatory network (GRN) was constructed following the standard procedures of the pySCENIC pipeline (Aibar et al., 2017). Co-expressing genes from the bin50 expression matrix with the human HUGO Gene Nomenclature Committee (hgnc) (Bruford et al., 2020) transcription factors (TFs) were identified using the GENIE3 algorithm (Huynh-Thu et al., 2010). To exclude false-positive results from the co-expression networks, the cisTarget database was then adopted to confirm the putative direct-binding targets of each TF, and filtered out the indirect targets lacking motif support. The verified co-expression modules were regulons with significant motif enrichment of the corresponding regulator. Finally, the AUCell algorithm was conducted to score the regulon activities of all bins and identify cell subgroups with high subnetwork activities (Aibar et al., 2017).

### Deconvolution: Cell2location

To identify cell type abundances in each spatial spot, we conducted deconvolution analysis by the cell2location software (Kleshchevnikov et al., 2022). A high-quality scRNA-seq reference panel is necessary for the accurate estimation of cell types in the spatial transcriptomic data, and here the scRNA-seq data from a previous study (Vento-Tormo et al., 2018) was adopted for the spatial data deconvolution. We selected the data sampled from placenta tissues and filtered out cells not belonging to SCT, VCT, EVT, FB, HB, and Endo for further analysis. First, the cell2location identified reference cell type signatures from the scRNA-seq profile using the negative binomial regression. Then, the cell-type gene-signature model was used to decompose the spatially resolved gene-count matrices into the reference cell-type signatures, thereby estimating the absolute cell abundance of individual cell types across all spatial spots.

### Cell-cell communication: CellPhoneDB

To study crosstalk between cell clusters mediated by ligand-receptor complexes, cell-cell communication analysis was performed using the CellPhoneDB software (https://github.com/Teichlab/cellphonedb). The CellPhoneDB datasets of ligands, receptors and their interactions were adopted, and the statistically significant enriched ligand-receptor interactions were identified based on the expression levels of ligands and coordinate receptors (Efremova et al., 2020).

## Results

### Comparison between the fresh and paraformaldehyde-fixed placenta villi

The Stereo-seq data of fresh and PFA-fixed treated villi were dramatically different in terms of tissue morphology and data quality. The cryosections of the fresh villi embedded in OCT showed poor morphology and changed tissue shapes, making it difficult to determine the structure. The H&E staining results of the sections showed that the outer SCT layer were ruptured. Many cells, especially the inner core cells beneath the SCT were lost (Supplementary Figure S1A). Approximate 1.8 G reads were generated in each section of the fresh sample with a Q30 bases of 89.5%. The number of gene types (mean gene type) detected in each bin50 area (37.5 μm × 37.5 μm) was 399 and the number of transcripts (mean molecular identifier, MID) detected in each bin50 was 820 (Supplementary Table S1). We observed strong diffusion of the transcriptome signals of the fresh villi from the sequencing results (Figure 1C), possibly due to RNAs spillover during the permeabilization of villi. In contrast, H&E staining of the PFA-fixed villi showed intact villi structures and good cell morphology, especially the integrity of the cells in the inner core of villi (Supplementary Figure S1B). Approximate 5.5 G reads were generated from the PFA fixed villi with a Q30 bases of 90.9%. The mean gene type of bin50 was 617 and the MID of bin50 was 1.59 K (Supplementary Table S1). Compared to the fresh villi, the sequencing results of the cryosections of the PFA-fixed villi showed dramatic reduction of the RNA signal diffusion (Figure 1D). Due to the severe RNA diffusion, trophoblast-specific markers could not be identified in the fresh villi (Supplementary Figure S2A). In contrast, Specific RNA signals of trophoblast-specific markers were identified in the PFA-fixed villi (Supplementary Figure S2B).

### Spatial transcriptome resolves the villi into distinct functional regions

**FIGURE 2:**
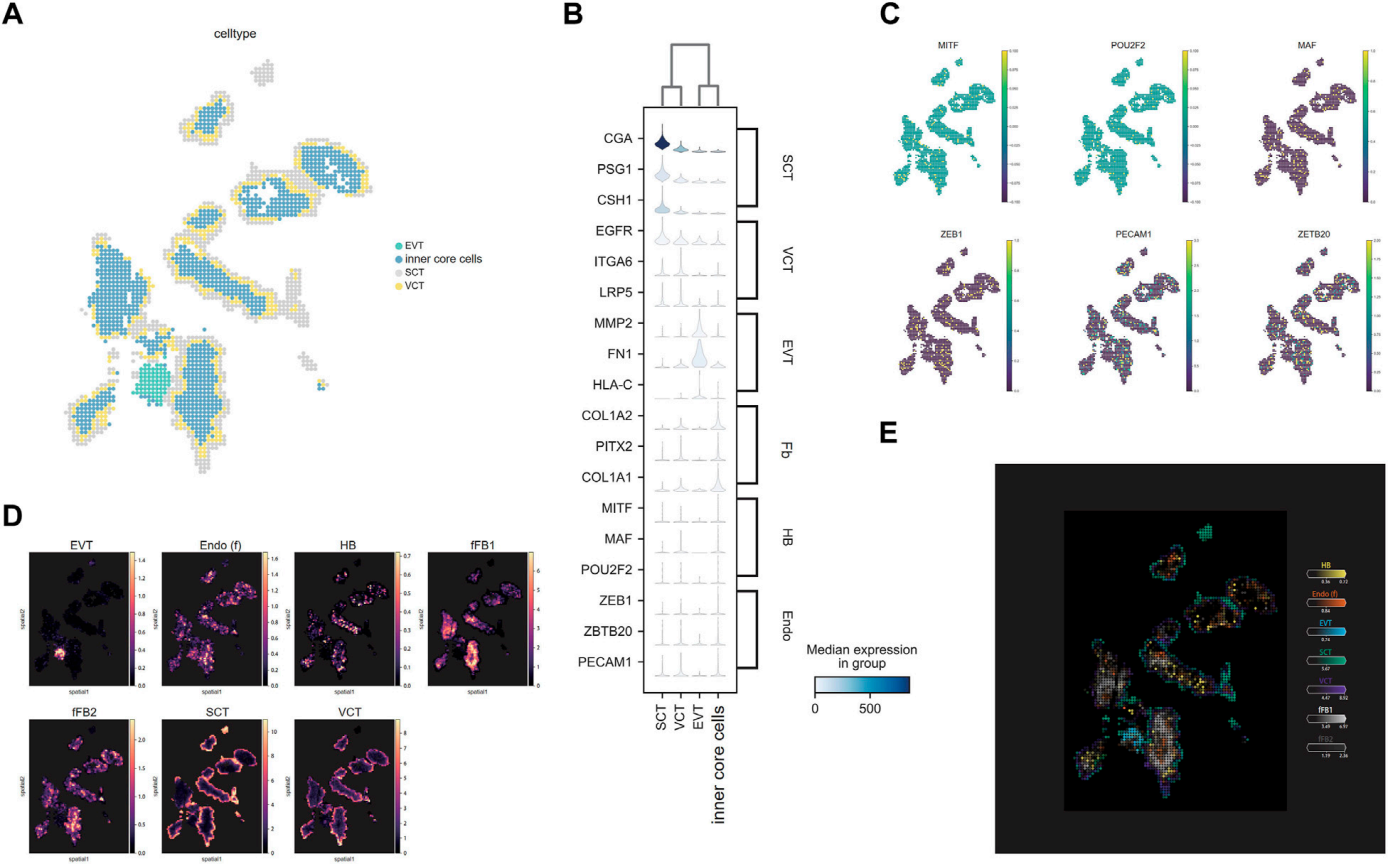
The localization of different cell types. **(A)** Unsupervised spatially constrained clustering of the villi. Bins are colored by their annotation. **(B)** The expression level and **(C)** the distribution of the markers for specific cell types in villi. **(D)** Estimated cell abundances (color intensity) of major cell types (color) across regions. **(E)** The distribution of cell types after deconvolution by cell2location showed in one map.

The PFA fixed villi were analyzed in detail for functional regions since they had better data quality and tissue morphology. A series of quality control (QC) steps were performed to remove background noise and low-quality bins that were mainly composed of mitochondrial and ribosomal RNAs. After data QC, the cell clusters corresponded well to the ssDNA image (Supplementary Figure S3). After cell bin clustering by SCC and cell type annotation, four major cell clusters were identified using the classic markers of placenta cells. To demonstrate the fine structure of a villus, a chip region with a representative integral villi structure was selected for detailed spatial transcriptome analysis (Figures 1D, 2A). Markers of SCT namely, *CGA* (Vento-Tormo et al., 2018), *PSG1* and *CSH1* (Sheridan et al., 2021) were identified in the outermost layer markers; VCT were identified in the inner layers of SCT using *EGFR* (Fock et al., 2015), *ITGA6,* and *LRP5* (Shannon et al., 2022) as markers; markers of EVT (*MMP2*, *FN1*, and *HLA-C*) (Papuchova et al., 2019) were identified in an anchoring villus-like cell column structure (Figure 2B). Besides trophoblasts, markers of FB including *COL1A1*, *COL1A2* and *PITX2* (Vento-Tormo et al., 2018) were identified in the core of the villi (Figure 2B). We also identified the markers of HB cells (*MTF*, *MAF* and *POU2F2*) and Endo cells (*ZEB1*, *ZBTB20* and *PECAM1*) (Papuchova et al., 2019) in the core of villi (Figure 2C). Since the FB, HB and Endo were mixed in the core of villi, we denoted them as the inner core cells. All the cell clusters were then deconvoluted by the Cell2location using the RNA-seq data from a previous study (Vento-Tormo et al., 2018). The results suggested that the HB and the Endo cells were dispersed among the FBs (Figure 2D). A localization map of different cell types in villi is shown in Figure 2E. Applying the same methodology, similar cell types and spatial localizations were found in the PFA-fixed villi of another chip (Supplementary Figure S4).

Gene ontology (GO) annotation was used to find the DEG and the involved biological processes in different trophoblast (Supplementary Figure S5; Supplementary Table S2). The most significantly upregulated GO biological processes in EVT versus VCT included cell migration, cell adhesion and cytoskeleton, while the pathways of cell proliferation such as protein translation, folding and negative regulation of degradation were downregulated in EVT. This result was in accordance with the previous data (Apps et al., 2011). Compared to VCT, the most significantly upregulated GO pathways in SCT included regulation of protein transport, hormone secretion, negative regulation of immune system process, viral entry into host cell, response to ER stress and apical plasma membrane, whereas the positive regulation of growth pathways was downregulated.

### Cell communication in villi predicted by CellphoneDB

**FIGURE 3:**
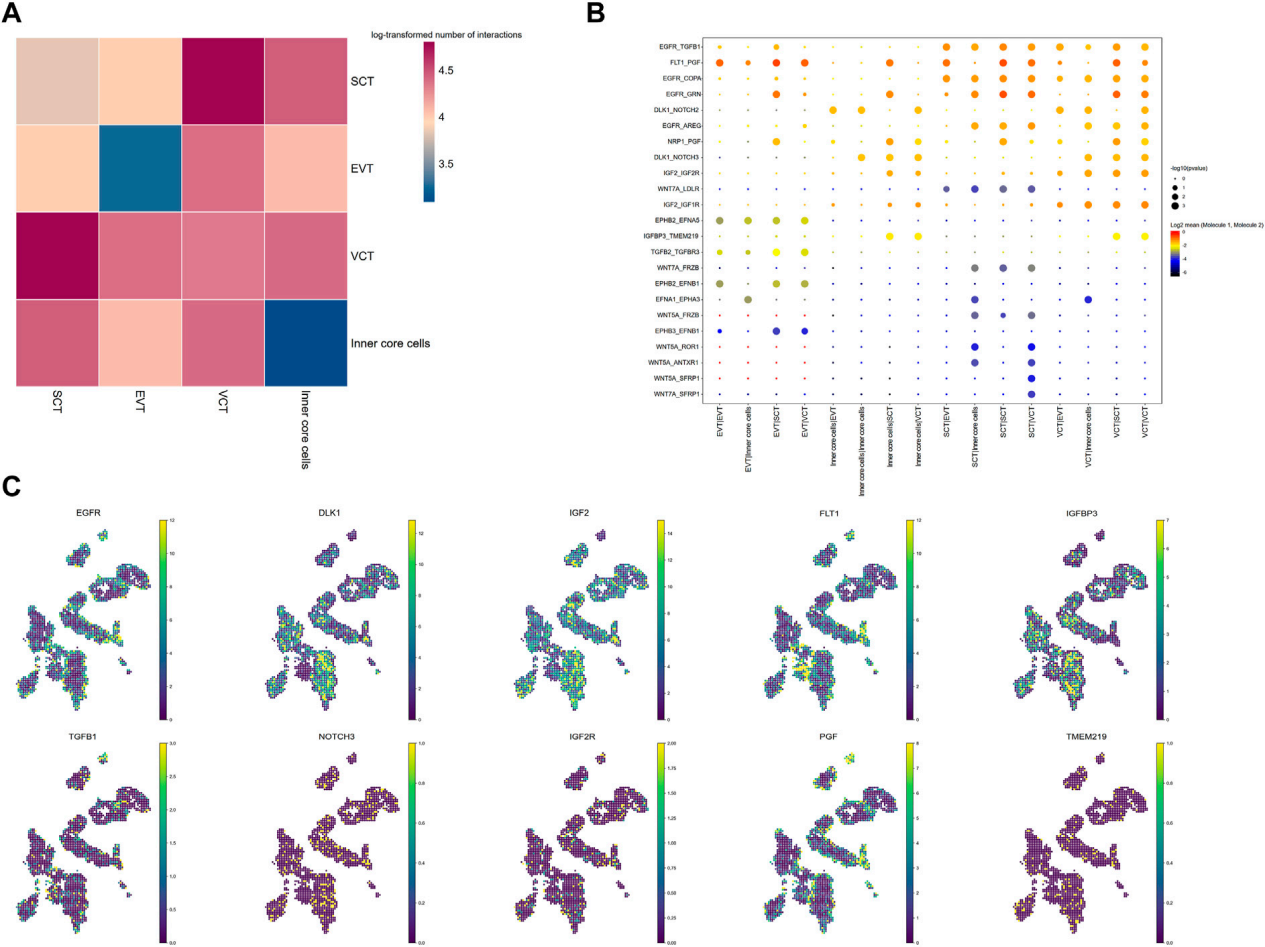
Intercellular interactions between different cell types in placenta villi. **(A)** Heatmap for the log-transformed number of ligand-receptor pairs in all cell clusters. The different colors indicate log2 of the counts. **(B)** Interactions between selected ligand–receptor pairs; *p* values indicated by the size of circles. The means of the average expression level of the molecules in ligand-receptor pairs are indicated by color. **(C)** The distribution of representative ligand-receptor pairs.

To investigate cell communications, we analyzed the expression level of ligand-receptor pairs in different cell types of the villi. SCT and VCT exhibited the highest number of ligand-receptor pairs, while the inner core cells have the least number of ligand-receptor pairs (Figure 3A; Supplementary Table S3). As expected, the ligand *DLK* localized mainly to FB, while its receptors, *NOTCH2* and *NOTCH3*, were expressed in all trophoblasts, implying a role of the interaction in maintenance of FB through paracrine signaling from trophoblast (Figure 3B). The interaction between *PGF* and *FLT1* was abundant in villi, both expressed in nearly all trophoblasts. The imbalance of this interaction is an indicator for PE (Maynard et al., 2003). The imprinted *Igf2*-*Igf2r* pairs have been well studied. In mice, Igf2 is expressed highly in feto-placental endothelial cells, and is important in maintaining trophoblast development, particularly the SynT-II layer (Sandovici et al., 2022). Our study found that *IGF2* was abundant in FB and VCT, while *IGF2R* was in all cell types. *Wnt* ligands such as *Wnt5A* and *WNT7A* were highly expressed in SCT, while the receptors of *Wnt*, including *FRZB* and *LDLR*, were expressed in SCT and inner core cells (Figure 3B). Wnt induces SynT-II cells fusion in mice through induction of *Gcm1* and *SynB* expression (Zhu et al., 2017). Here, Wnt may function on SCT through an autocrine action. *EGFR* was also highly expressed in SCT and VCT, while the ligands were expressed in nearly all the villous cells (Figure 3B). The expression of EGFR increases with differentiation of the SCT (Rab et al., 2013). With the Stereo-seq data, we directly mapped the location of various ligand receptors in the villi, and showed their co-localization with corresponding ligands inferring the physical contact between specific cells with ligand-receptor pairs (Figure 3C).

### Spatial regulatory activity of transcription factors in villi

**FIGURE 4:**
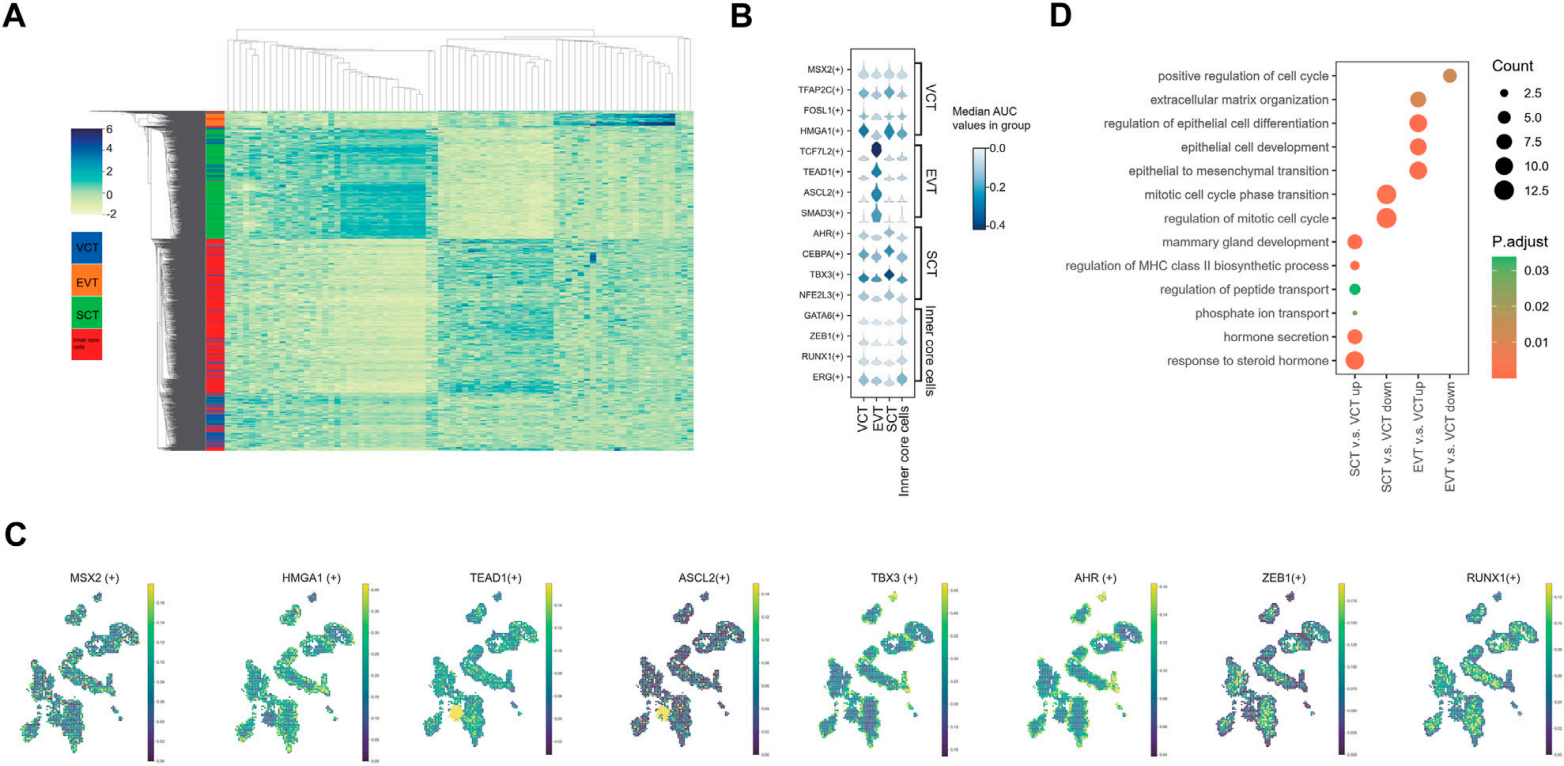
Analysis of the spatial regulatory activity of transcription factors in villi. **(A)** Heatmap of AUC Z-scores for the cell-type-specific regulons **(B)** The AUC score levels of representative regulons. **(C)** Distribution of the representative regulons. **(D)** GO pathway analysis of differentially expressed regulons.

Using the SCENIC analysis, we identified a total of 316 regulons in EVT, VCT, SCT and inner core cells. The top 20 regulons specific in each cell type were identified based on the regulon specificity score (RSS). The heatmap generated from the Z-scores of these cell-type-specific regulons shows that the cell clusters based on the regulons AUC scores (Figure 4A; Supplementary Table S4). A group of TFs reported to be specifically expressed in the placenta was used to confirm our analysis of spatial regulatory activity (Figure 4B). For example, *TFAP2C*, *MSX2, FOSL1* and *HMGA1* are expressed in VCT (Renaud et al., 2014; Hornbachner et al., 2021). We showed consistent findings on the expression and spatial localization of these TFs (Figures 4B,C). However, we did not find the expression of *FOSL1* and *HMGA1* in the EVT, which is against the report that *FOSL1* is also expressed in the proliferative EVT cell columns, while *HMGA1* in the EVT invading the decidua (Renaud et al., 2014). *TCF7L2, TEAD1, ASCL2,* and *SMAD3* have been reported to be expressed specifically in EVT (Meinhardt et al., 2014; Dong et al., 2022; Haider et al., 2022). These TFs were also highly expressed in EVT in our results (Figure 4B). We also confirmed the dominant expressions and spatial distribution of *AHR, CEBPA, TBX3 and NFE2L3* in the nuclei of SCT (Figures 4B,C), consistent with previous studies (Simmons et al., 2008; Iqbal et al., 2021; Chen et al., 2022b; Dong et al., 2022). *GATA6, ZEB1, RUNX1, and ERG* are fibroblast or endothelial cells-related TFs (Molkentin, 2000; Ponder et al., 2016; Yuan et al., 2020; Zhang et al., 2021b; Caporarello et al., 2022). We confirmed that these TFs were mostly expressed in the inner core cells (Figures 4B,C). To further validate our results, we acquired the protein expression data of the above TFs from the Human Protein Atlas (https://www.proteinatlas.org/), which showed the concordant expression in cell types and localization (Supplementary Figure S6).

By GO analysis, the regulons upregulated in SCT relative to VCT were related to hormones transport and secretion, and response to hormones (Figure 4D; Supplementary Table S5). This is consistent with the fact that the SCT synthesize and secrete a variety of hormones, including hCGA and hCGB, CSH1, PSGs, PGF, CRH and others (Jaremek et al., 2021). Meanwhile, the regulons associating with the regulation of mitotic cell cycle were downregulated, which is concordant with the exit from the mitosis before differentiation of SCT. Pathways of nutrient transport and mammary gland development were also identified (Figure 4D). In EVT, pathways such as the epithelial to mesenchyme transition pathway, epithelial development and differentiation, extracellular matrix organization were identified as previously described (Illsley et al., 2020; Papuchova and Latos, 2022), which were associated with the invasive activities of EVT (Figure 4D; Supplementary Table S5).

### The expression of virus receptors and drug transporters in syncytiotrophoblasts

**FIGURE 5:**
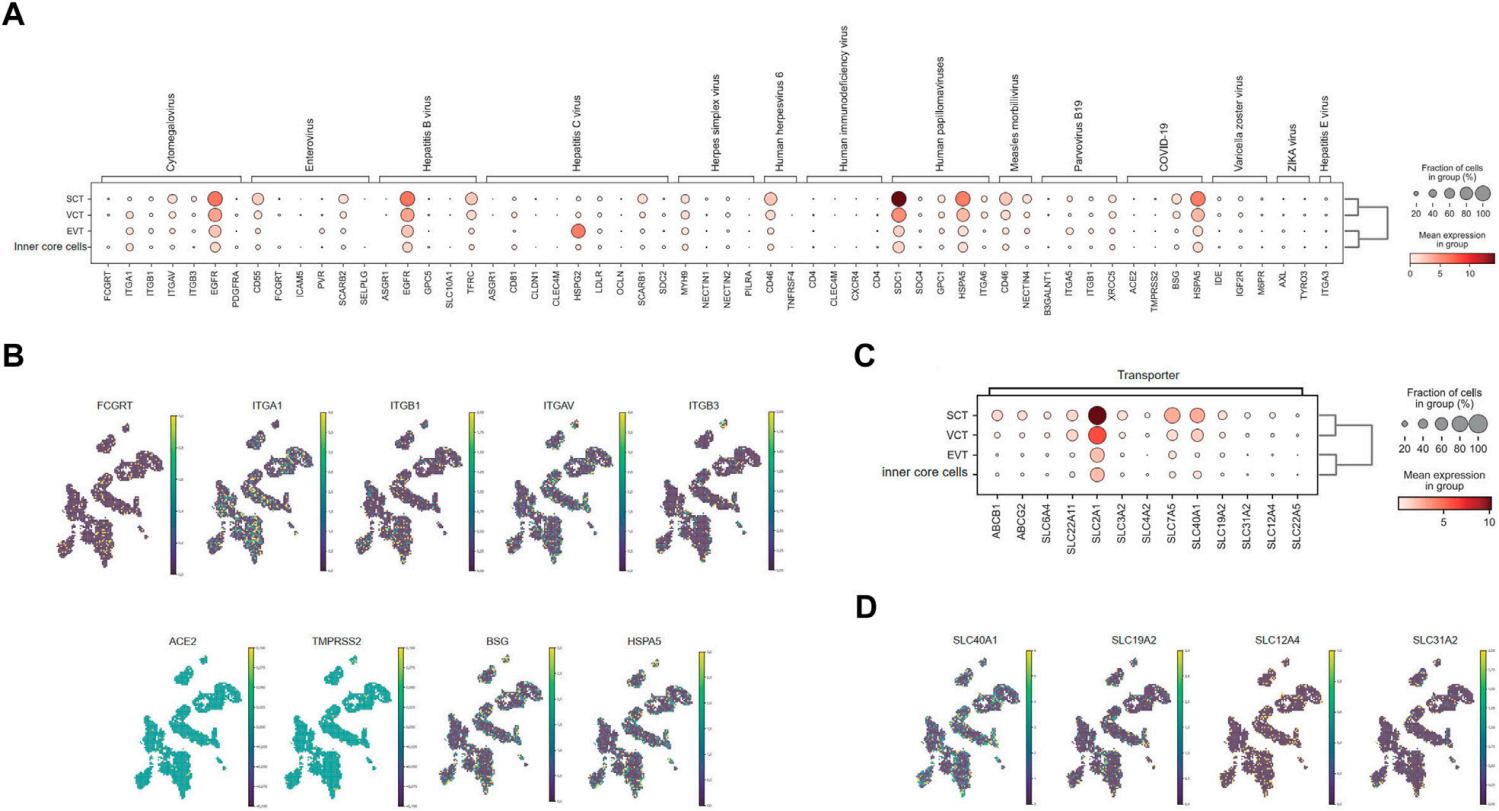
Distribution of virus receptors and drug transporters in chorionic villi. **(A)** Expression level of receptors for different viruses in villi. **(B)** Distribution of representative virus receptors. **(C)** Expression level of representative drug transporters in villi. **(D)** Distribution of representative drug transporters.

As the outermost cell type, SCT has direct contact with the maternal interface. We found that the DEG of SCT contained the genes involving in viral entrance to cells and viral life cycles, implying possible vertical transmission of virus from mother to fetus through SCT (Supplementary Figure S7; Supplementary Table S6). To evaluate the placental receptors of virus transmission, we mapped the expression of a panel of 20 viral receptors and co-receptors for TORCH (Toxoplasma gondii, Other agents, Rubella, Cytomegalovirus & Herpes simplex virus), severe acute respiratory syndrome coronavirus 2 (SARS-CoV-2), ZIKA, and other infectious diseases in the villi (Figure 5A). Generally, *EGFR, SDC1, HSPA5, MYH9, CD46* were strongly expressed in the SCT but also widely expressed in other cell types. In contrast, *ITGAV, CD55, TFRC, SCARB1, HSPG2,* and *BSG* were only mainly expressed in SCT.

Human cytomegalovirus (HCMV) may infect SCT to induce monocyte activation through the receptor EGFR and the co-receptor integrin proteins (Wang et al., 2003; Chan and Guilbert, 2005). However, this theory is still controversial, as the virion may across the syncytium by transcytosis instead of direct infection (Fisher et al., 2000; Maidji et al., 2006). We found that SCT exhibited little expression of the neonatal Fc receptor (*FCGRT*), which helps to transport IgG for passive immunity. Yet, the key integrin receptors, *α1β1* and *αVβ3*, together with *EGFR* had moderate or strong expression in SCT (Figures 5A,B), which provides the molecular basis for viral infection. The protein expressions of α1β1 and αVβ3 in SCT were also verified by the data from the Human Protein Atlas (Supplementary Figure S8).

Till now, it is still controversial whether the SARS-CoV-2 can be vertically transmitted (Islam et al., 2020; Rodrigues et al., 2020; Yoon et al., 2020; Kotlyar et al., 2021). We assessed the expression of a series of SARS-CoV-2 receptors in villi. Our results indicated a low expression of *ACE2* and *TMPRSS2* in all cell types in the villi (Figures 5A,B), consistent with previous single-cell analysis studies (Lu et al., 2020; Pique-Regi et al., 2020). However, *BSG* and *HSPA5* were expressed highly in the SCT as previously reported (Dong et al., 2021; Lee et al., 2022) (Figures 5A,B), inferring the possibility of vertical transmission of SARS-CoV-2.

In SCT, the transporter genes were strongly upregulated, inferring their roles in transportation of nutrition, gas, and hormones between mother and fetus (Supplementary Figure S7; Supplementary Table S6). Specifically, we analyzed the transporter genes relevant to drug transportation, including 52 members in ATP-binding cassette (ABC) family (Sarkadi et al., 2006) and 395 members in solute carrier protein (SLC) families (Schlessinger et al., 2013). A total of 47 transporters were enriched in SCT, most of which have been previously reported (Figure 5C; Supplementary Figure S9). *ABCB1*, for example, has been reported to localize at the apical membrane of SCT to back-transport xenobiotics and drugs to maternal circulation (Sun et al., 2006). *ABCG2* is also localized predominantly to the apical surface of SCT, and limits the transfer of nitrofurantoin (Merino et al., 2005), cimetidine (Pavek et al., 2005) and glyburide (Gedeon et al., 2008) to fetal circulation. Moreover, *SLC6A4* (Wang et al., 2007), *SLC22A11* (Shimizu et al., 2005), *SLC2A1*, *SLC3A2*, *SLC4A2* (Nishimura and Naito, 2008), *SLC7A5* (Jia et al., 2022) protein or RNA have been shown to localize to SCT. These were all detected in our data (Figures 5C,D; Supplementary Figure S9). We also found transporters that had not been elucidated in the placenta, such as *SLC40A1*, *SLC19A2* and *SLC31A2* (Figure 5D; Supplementary Figure S10), which could be candidates for future study on blood-placental barrier.

## Discussion

Spatial transcriptome is important for understanding the structure-function relationship of placenta. For the first time, we provided the spatial transcriptome profile of chorionic villi of early placenta (8 weeks gestation) using our proprietary Stereo-seq method. The main cell types of placental villi were identified, and their spatial localizations were accurately pinpointed on the villi structure at single-cell level. Cell communication in the villi was identified and specific TFs were found to be involved in regulating differentiation and functions of trophoblast in villi. Finally, the viral receptors and drug transporters, which is important for the blood-placental barrier, were spatially identified in villi.

Human placenta is not a compact structure and is rich in extracellular matrix composing of syndecans and integral proteoglycans (Moore et al., 2021). We found that the loose structure of fresh placenta villi was incompatible with the Stereo-seq method, as shown by the severe RNA diffusion and damaged tissue morphology. We reckoned that the reason is due to cell damage during sectioning, leading to diffusion of intracellular RNA. In comparison, PFA fixation of placenta villi distinctly reduced RNA diffusion and improved the specificity of signal localization. Although PFA fixation may cause cross-linking of nuclei acid and damage RNA integrity, sufficient gene numbers in each cell bin were obtained by increasing the sequencing depth. Hence, we recommend the PFA fixation of placenta for spatial transcriptome profiling of the first-trimester placenta villi by Stereo-seq. It is worth to verify the usefulness of PFA fixation on other samples with complex but loose structures, such as small intestinal villi, testis and lung.

Extensive works have been done to illustrate the cell types and cell interactions in placenta by scRNA-seq (Pavlicev et al., 2017; Perez et al., 2022). With our Stereo-seq data, we identified SCT, VCT, EVT and FB as the main cell types in the placenta villi, consistent with previous histological and scRNA-seq studies. Meanwhile, the placenta scRNA-seq data were used to reinforce our spatial transcriptome analysis. By deconvolution of the spatial expression matrix with scRNA-seq data, we revealed the localization of two minor cell types of HB and Endo in villi. Functioning as fetal macrophages, HB plays important roles in anti-pathogens, placental vasculogenesis, angiogenesis and SCT differentiation (Seval et al., 2007). Endo are the main cell type of blood vessels in the villi that can interact with HB to regulate growth of the vessels. The stereo-map colocalized these two cell types and demonstrated their interaction.

A major advantage of spatial transcriptome comparing with scRNA-seq is the localization of specific cell subtypes, and determination of cell functions, cell interaction and microenvironment in spatial context. In this study, we demonstrated the usefulness of Stereo-seq data in studying the ligand-receptor pairs and spatial regulatory activity in villi cells. Using the CellPhoneDB, we confirmed the previously found ligand-receptor pairs including *PGF*-*FLT1* (Maynard et al., 2003), *DLK*-*NOTCH3* (Cleaton et al., 2016) in different cell types in the villi. We further identified new pairs, including the *IGF2*-*IGF2R* and the *WNT*-*FRZB* pairs, providing new aspects for the study of villi development. Although previous analyses of CellphoneDB were mainly based on scRNA-seq data, our results show that it is also applicable to our spatial transcriptome data. The proximity is one of the most important determining factors of cell-to-cell interactions. For paracrine or membrane-bond ligands, interaction exists between neighboring cells. We used SCENIC to construct GRN and found four main modules, which were consistent with the localization of different cell subtypes, and further verified the reliability of the Stereo-seq data. GO analysis of TFs also indicated the developmental dynamics during VCT differentiation towards EVT and SCT, and reflecting the function of placenta, including pregnancy hormone production, invasion to decidua to remodel the spiral artery.

The Stereo-seq data can also be used to analyze the distribution of viral receptors and transporters in placental cells. We found that the receptors of HCMV and SARS-CoV-2 were expressed in cells in villi including SCT. Our results provided some theoretical basis for predicting mother-to-fetus transmission of the virus and guiding medication during pregnancy. In this study, we showed some potential application scenarios of spatial transcriptomics on placenta villi, including hormone secretion, nutrient and gas exchange, decidua remodeling, and the developmental regulation of villi. However, we could not study the pathways of immune response because there was no decidua involved in this study, which is the main region where feto-maternal immune response occurs.

In this study, the spatial transcriptome analysis was conducted with the resolution of bin50, which approximately equals to an area of 37.5 μm × 37.5 μm. As multiple cells may be present in the area, a true single-cell profile of placenta villi was not obtained in this study. The lack of the spatial transcriptome profile of placenta villi at single-cell level may explain why it needed to use the deconvolution method to identify rare cell types in the villi, such as HB and Endo mixed with FBs. It is possible to further improve the resolution to cell bin20 or above by increasing the sequencing depth to obtain a higher gene number for each cell (Wang et al., 2022). Another possible strategy is to determine the cell border to perform the spatial single-cell analysis *via* bioinformatic approaches (Wei et al., 2022).

## Conclusion

In this study, we successfully constructed the spatial transcriptome of the early placenta villi using a modified method that may be applied to tissues with a loose structure. All the main cell types in the villi were identified in their physiological position. We investigated the regulons and found specific TFs in different cell types associated with the differentiation and functions of trophoblasts. Finally, the viral receptors and drug transporters in villi were identified according to the pathway analysis. Our work provides a valuable data resource for further investigation of the placenta structure, development, physiology and pathology.

## Data Availability

The original contributions presented in the study are included in the article/Supplementary Materials, further inquiries can be directed to the corresponding authors.
