# Supplementary material for: Resolving the gene expression maps of human first-trimester chorionic villi with spatial transcriptome
Source: Front Cell Dev Biol. 2022 Dec 6;10:1060298. doi: 10.3389/fcell.2022.1060298 (PMC9763897; doi:10.3389/fcell.2022.1060298)
Supplement: Supplementary file 4 [file DataSheet1.PDF]

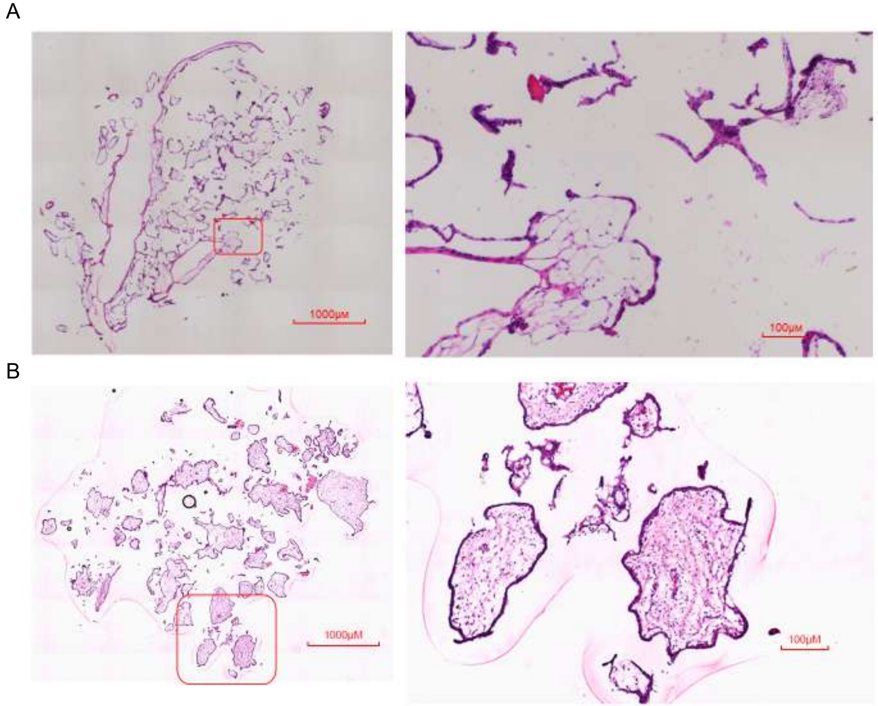

Figure S1. H&E staining of fresh or PFA-fixed chorionic villi. (A) H&E staining of fresh villi. (B) H&E staining of PFA-fixed villi.

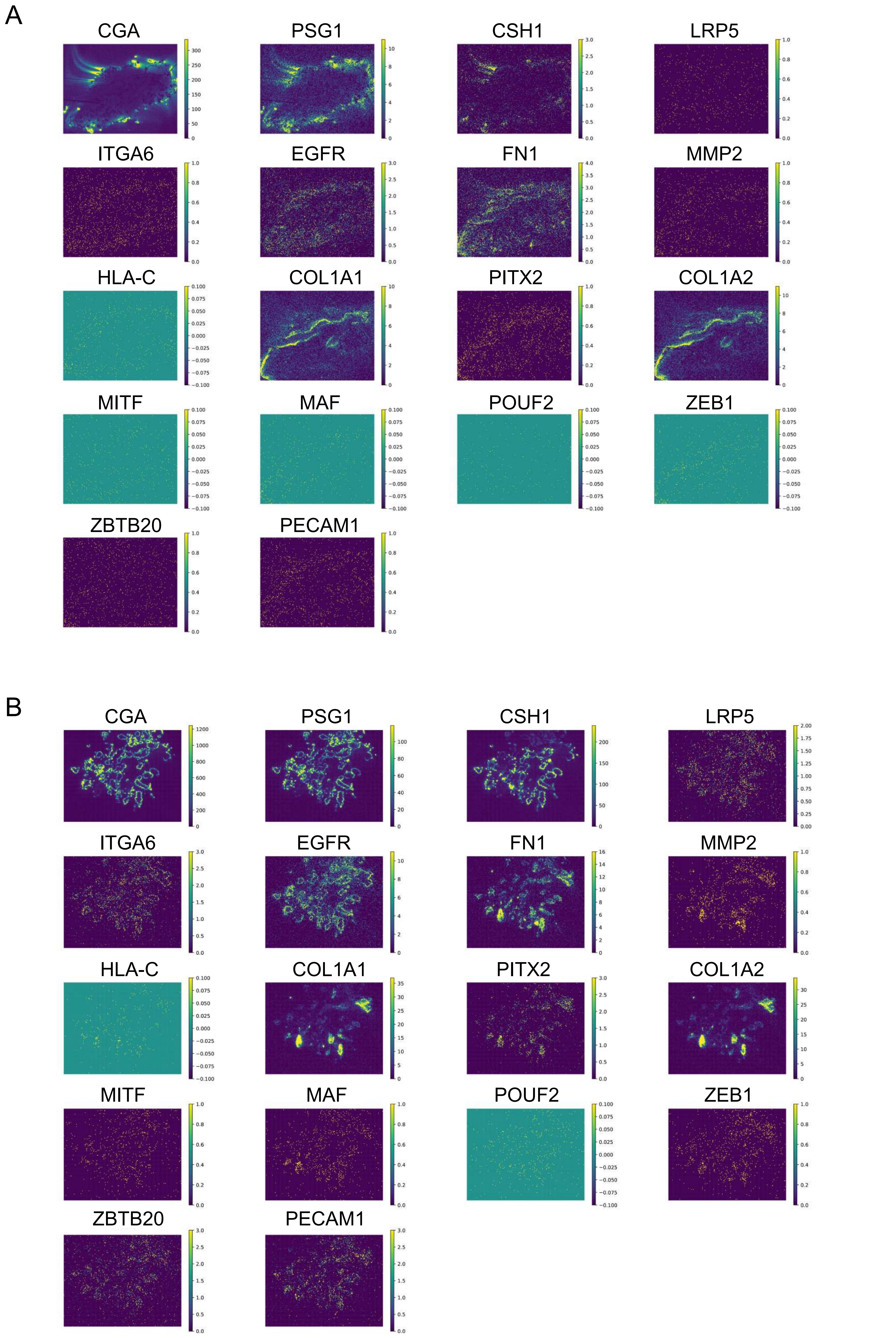

Figure S2. Density of the signals of representative markers in chips of fresh or PFA-fixed villi. (A) Density of the signals of markers in chips of fresh villi. (B) Density of the signals of markers in chips of PFA-fixed villi.

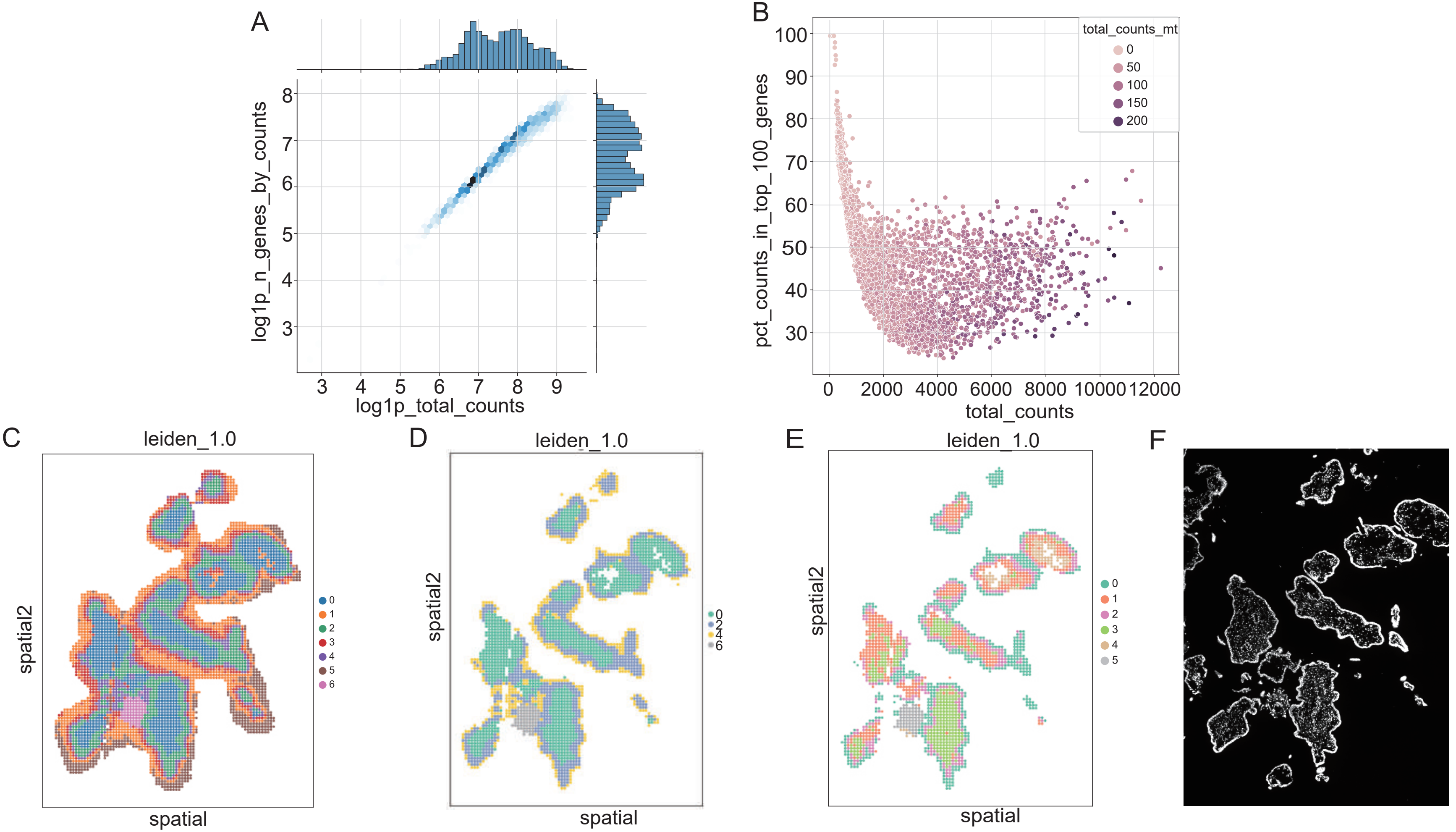

Figure S3. The workflow to filter the pseudo-positive signals. (A) The bimodal distributions of both log1p total counts and log1p n genes by counts. (B) bin50s with the percent counts in top 100 genes. (C) The remaining bins were clustered by using SCC. (D) Wilcoxon test was conducted to identify statistically significant DE genes. (E) The final bin50 count matrix after filtering out the low-quality cluster 4. (F) ssDNA staining of the indicated area on the chip.

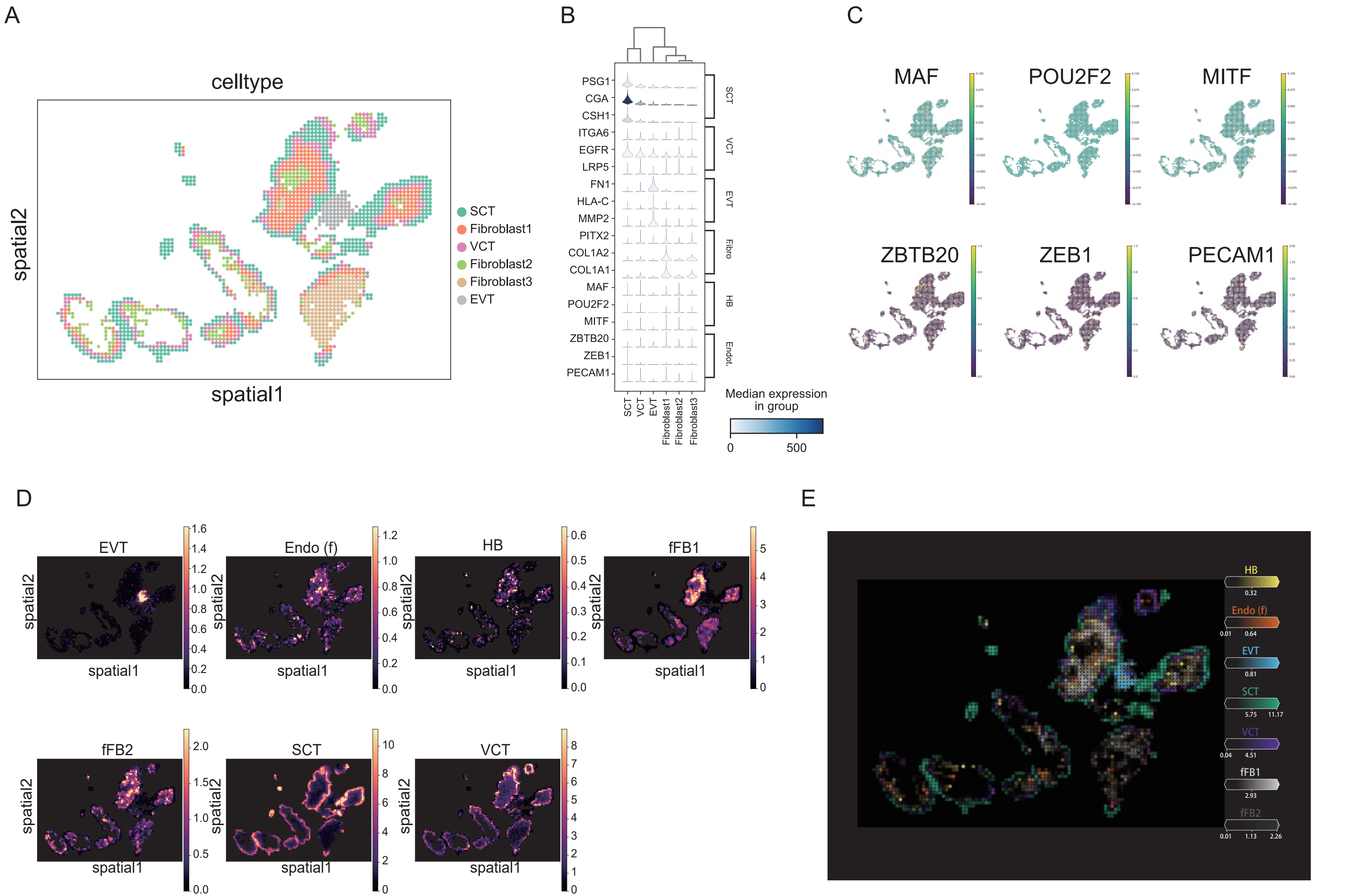

Figure S4. The localization of different cell types in the adjacent slide. (A) The localization of cells identified by SCC. (B) The expression level of the markers for specific cells in villi. (C) the distribution of the markers in (B). (D) The distribution of cells after deconvolution by cell2location. (E) The distribution of cells after deconvolution by cell2location showed in one map.

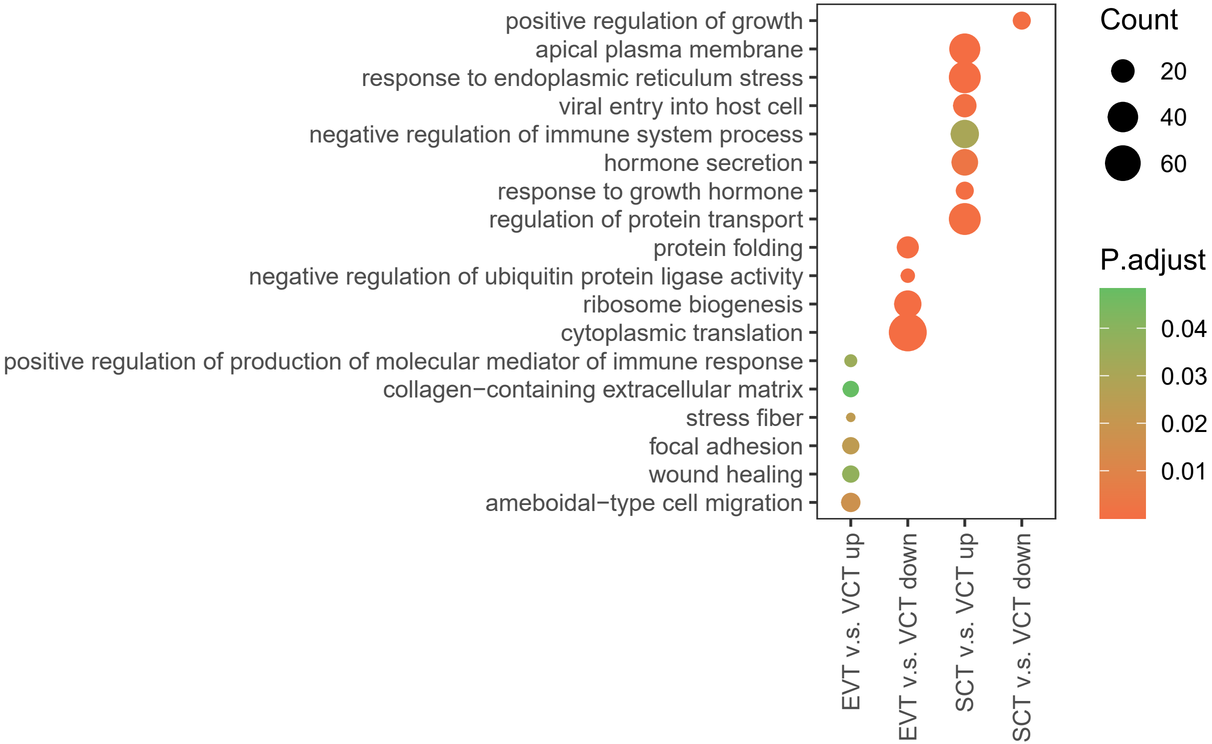

Figure S5. GO pathway analysis of differentially expressed genes in different trophoblasts.

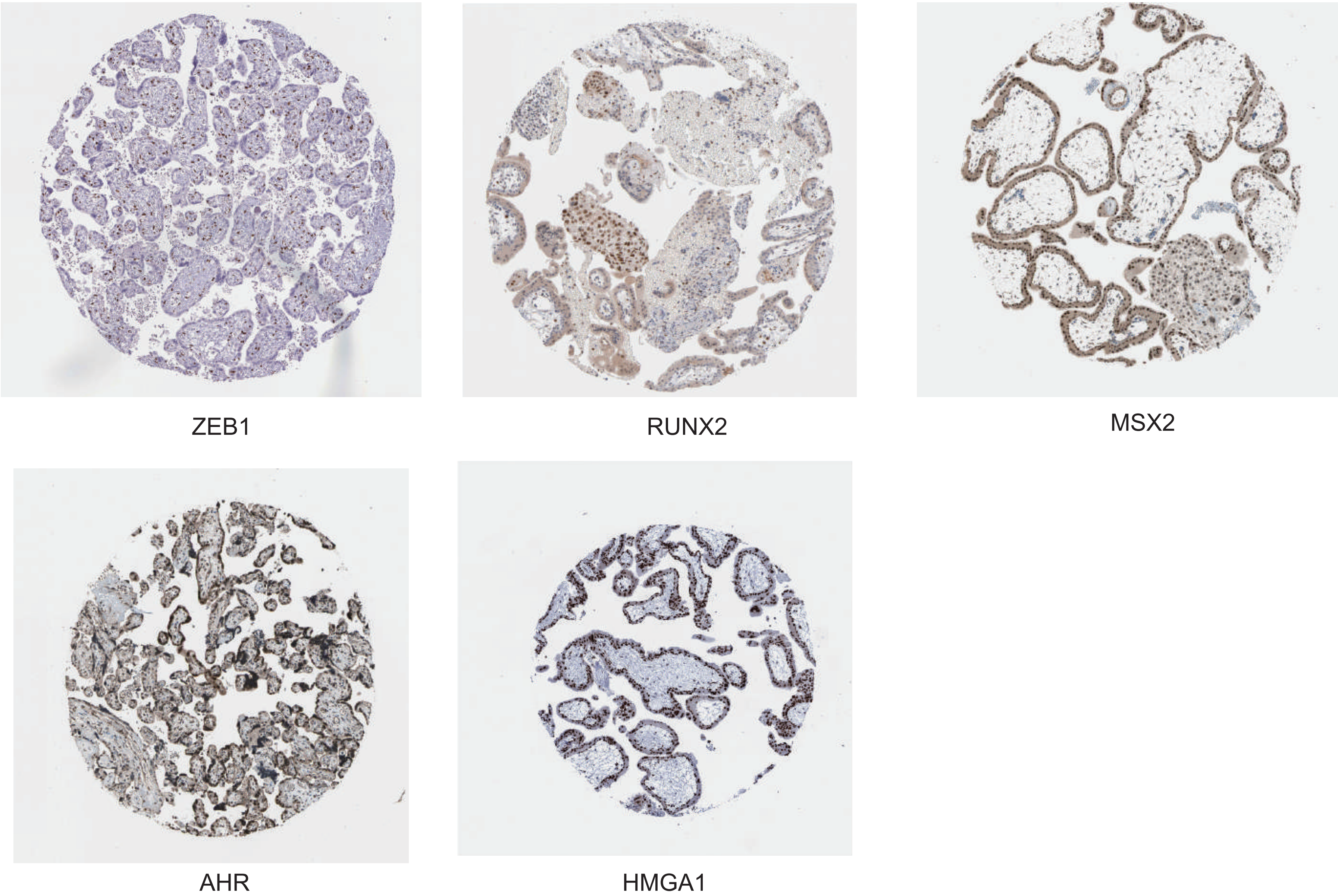

Figure S6. The localization of the proteins of representative transcription factors in placenta. The figures were downloaded from HPA (https://www.proteinatlas.org/).

f

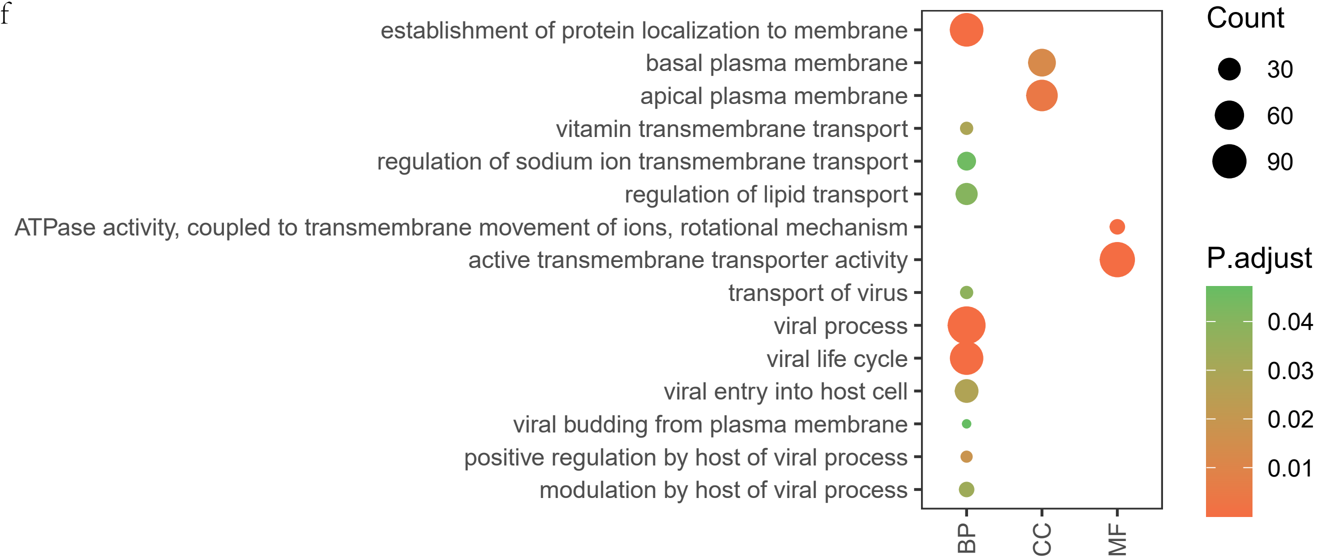

Figure S7. The GO pathway analysis using the DEGs up-regulated in SCT versus other cell types.

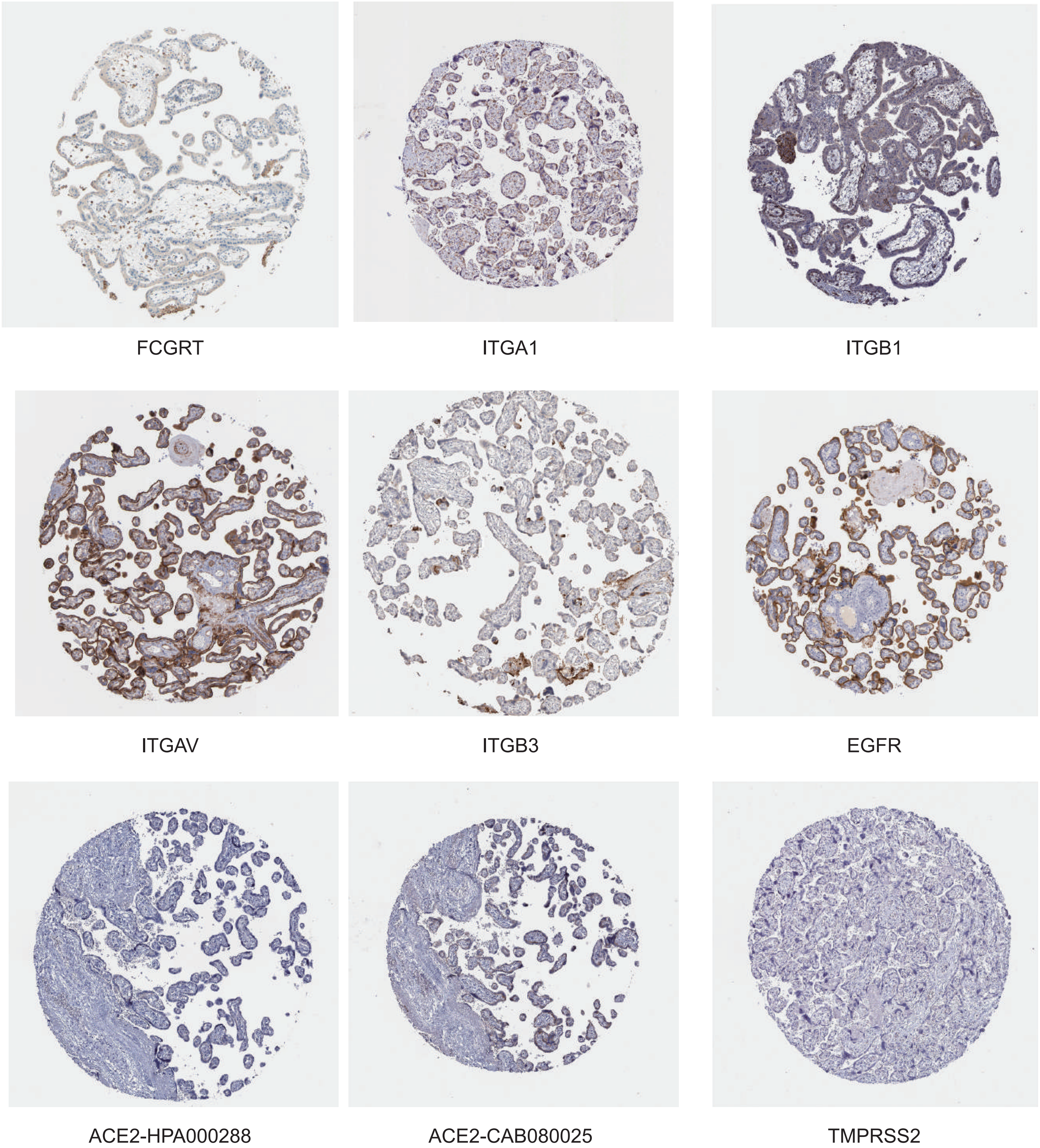

Figure S8. The localization of the proteins of representative virus receptors in placenta. The figures were downloaded from HPA (https://www.proteinatlas.org/).

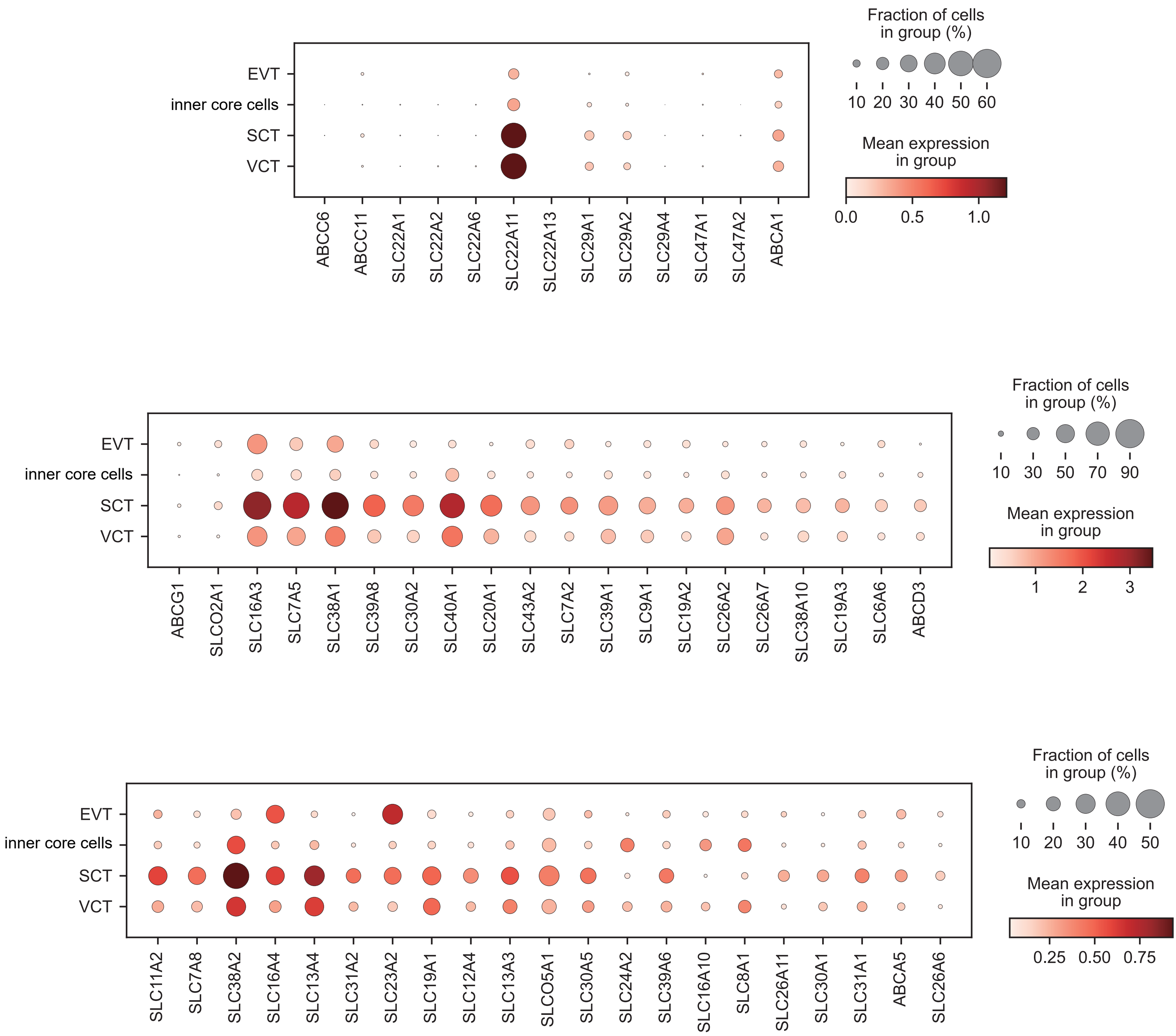

Figure S9. The expression level of drug transporters enriched in SCT.

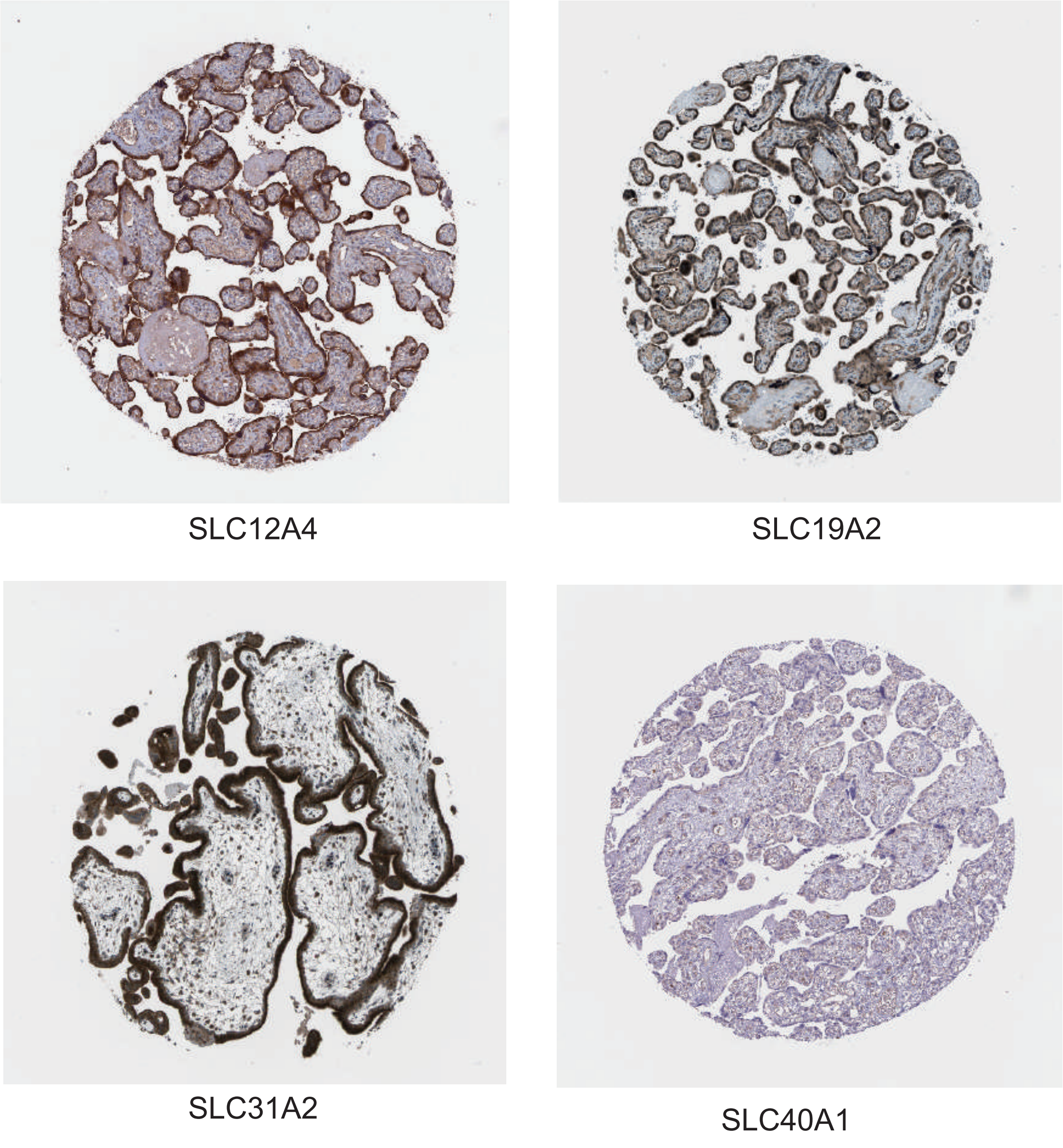

Figure S10. The localization of the proteins of representative drug transporters in placenta. The figures were downloaded from HPA (https://www.proteinatlas.org/).
